# Neuronal Sirt3 Protects against Excitotoxic Injury in Mouse Cortical Neuron Culture

**DOI:** 10.1371/journal.pone.0014731

**Published:** 2011-03-01

**Authors:** Sun Hee Kim, Hua Fei Lu, Conrad C. Alano

**Affiliations:** 1 Department of Neurology, Veterans Affairs Medical Center, San Francisco, California, United States of America; 2 Department of Neurology, University of California, San Francisco, California, United States of America; University of North Dakota, United States of America

## Abstract

**Background:**

Sirtuins (Sirt), a family of nicotinamide adenine nucleotide (NAD) dependent deacetylases, are implicated in energy metabolism and life span. Among the known Sirt isoforms (Sirt1-7), Sirt3 was identified as a stress responsive deacetylase recently shown to play a role in protecting cells under stress conditions. Here, we demonstrated the presence of Sirt3 in neurons, and characterized the role of Sirt3 in neuron survival under NMDA-induced excitotoxicity.

**Methodology/Principal Findings:**

To induce excitotoxic injury, we exposed primary cultured mouse cortical neurons to NMDA (30 µM). NMDA induced a rapid decrease of cytoplasmic NAD (but not mitochondrial NAD) in neurons through poly (ADP-ribose) polymerase-1 (PARP-1) activation. Mitochondrial Sirt3 was increased following PARP-1 mediated NAD depletion, which was reversed by either inhibition of PARP-1 or exogenous NAD. We found that massive reactive oxygen species (ROS) produced under this NAD depleted condition mediated the increase in mitochondrial Sirt3. By transfecting primary neurons with a Sirt3 overexpressing plasmid or Sirt3 siRNA, we showed that Sirt3 is required for neuroprotection against excitotoxicity.

**Conclusions:**

This study demonstrated for the first time that mitochondrial Sirt3 acts as a prosurvival factor playing an essential role to protect neurons under excitotoxic injury.

## Introduction

Continuous supply of energy is crucial for neuron survival due to the requirement for large amounts of energy for high metabolic processes coupled with an inability to store energy [1], [2]. Therefore, neurons are highly susceptible to insults that lead to energy depletion, such as oxidative stress, excitotoxicity, and DNA damage [3], [4]. As a critical factor in energy metabolism for cell survival, nicotinamide adenine dinucleotide (NAD) has drawn considerable interest. NAD is an essential molecule playing a pivotal role in energy metabolism, cellular redox reaction, and mitochondrial function. Recent studies have revealed that maintaining intracellular NAD is important in promoting cell survival in various types of diseases, including axonal degeneration, multiple sclerosis (MS), cerebral ischemia, and cardiac hypertrophy [5], [6], [7], [8], [9], [10], [11], [12]. Loss of NAD decreases the ability of NAD dependent cell survival factors to carry out energy dependent processes, leading to cell death.

PARP-1, a major NAD metabolizing enzyme, hydrolyzes NAD to nicotinamide and produces poly (ADP) ribose polymers (PAR) upon activation under pathological condition, and leads to severe impairment of energy metabolism with almost complete depletion of cytosolic and nuclear NAD [13], [14], [15], [16]. Genotoxic injury, overstimulation of *N*-methyl-D-aspartate (NMDA) receptors, or oxidative stress has been reported to activate PARP-1 in neurons [17], [18]. PARP-1 mediated NAD depletion results in mitochondrial permeability transition (MPT) causing cell death under DNA damaging injury [19], [20]. Our recent study showed that NAD depletion is a causal event in PARP-1-mediated cell death in astrocytes [19], neurons [21], and cardiac myocytes [22].

The silent information regulator 2 (SIR2) is a family of proteins with NAD-dependent deacetylase activity highly conserved from bacteria to humans [23], and is required for gene silencing and life span extension [24], [25], [26], [27]. Seven isoforms of the mammalSir2 homolog, sirtuin (Sirt) 1–7, are known to be expressed in mammals [28]. As broader ranges of substrates were found, many cellular functions of Sirts have been revealed, including senescence, apoptosis, energy metabolism, and stress resistance [29], [30], [31], [32], [33]. Sirt1 serves as a founding member of this family, and evidence of its role in neuronal death has recently been reported. Overexpression of Sirt1 and the use of the Sirt1 agonist, resveratrol, were neuroprotective against amyloid beta toxicity by reducing microglial NF-kappaB signaling [34]. Sirt1 was shown to protect neurons against neurodegeneration in Alzheimer's disease and amyotrophic lateral sclerosis [35]. Sirt3 was the only sirtuin implicated in extension of life span in human [36], and recent evidence have shown its involvement in mitochondrial energy metabolism and biogenesis [37] and preservation of ATP biosynthetic capacity in the heart [38]. Sirt3 was shown to regulate the activity of acetyl-CoA synthetase 2 (AceCS2), an important mitochondrial enzyme involved in generating acetyl-CoA for the tricarboxylic acid (TCA) cycle. In these studies, Sirt3 knockout resulted in a marked decrease of basal ATP level in vivo [39]. Recent studies in cardiomyocytes demonstrated the protective role of Sirt3 from oxidative stress and hypertrophy [11], [40]. Accordingly, these evidences suggest that Sirt3 could also have a pivotal role in protecting neurons from injury due to conditions that promote bioenergetic failure, such as excitotoxicity.

In this study, we demonstrated the presence of Sirt3 in neurons, and explored the role of Sirt3 in protecting neurons against excitotoxic injury. We found that Sirt3 expression was increased in mitochondria following PARP-1 mediated NAD depletion under excitotoxic injury. By modulating Sirt3 levels in neurons using either overexpression or knockdown, we provided evidence to show that Sirt3 contributes to neuronal protection by suppressing oxidative stress. This study provides the first evidence that Sirt3 plays an important role in neuronal survival against an excitotoxic insult.

## Results

### NAD depletion causes neuronal death with excitotoxic injury

**Figure 1:**
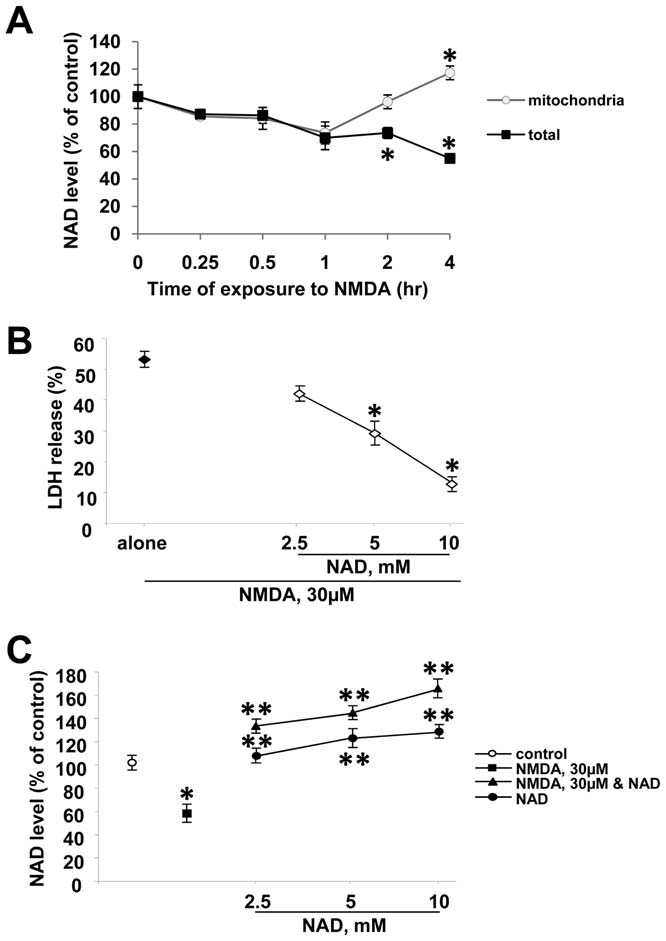
NAD depletion underlies excitotoxic neuronal death. (A) NAD level was measured in total cellular and mitochondrial fractions in mouse primary cortical neuron cultures exposed to NMDA 30 µM for indicated times. (mean ± SEM; n = 8; *P<0.01, difference from untreated control). (B) Cell death was assessed by measurement of LDH release (mean ± SEM; *n* = 8 ; *P<0.01, difference from NMDA alone). (C) NAD level was measured in neurons 4 h after exposure to NMDA 30 µM alone, NMDA plus various doses of NAD, or NAD alone (mean ± SEM; n = 4; *P<0.01, difference from untreated control; **P<0.01, difference from NMDA alone).

Excitoxicity induces energy failure by increased consumption of ATP, during which process NAD, an important factor in energy metabolism, rapidly drops [9], [17]. First, we examined the effect of NMDA receptor activation on NAD levels upon prolonged exposure to NMDA that leads to ∼50% death [41], [42]. NAD was measured in both total cell fraction and mitochondria fraction of primary neuron cultures treated with 30 µM NMDA. Consistent with our previous study showing the selective depletion of cytosolic NAD following PARP-1 activation under genotoxic stress [20], [21], we observed a selective NAD depletion in the cytosol but not in mitochondria of neurons at the indicated time points after NMDA treatment (Fig. 1A). No cell death was found 4 h after NMDA exposure (data not shown). Prolonged exposure to NMDA induced nearly 50% neuronal death after 24 hours. To examine whether NAD depletion contributed to NMDA-induced neuronal death, we treated neurons with NMDA alone or NMDA with exogenous NAD, which we have previously shown to increase cellular NAD [21]. If loss of cellular NAD causes neuronal death, then treatment with exogenous NAD should be able to rescue neurons from prolonged exposure to NMDA. Treatment with exogenous NAD dose dependently rescued neurons by restoring cellular NAD levels (Fig. 1B and 1C), suggesting that loss of cellular NAD levels is the cause of neuronal death. NAD treatment with NMDA led to a slightly higher NAD level than NAD alone, though not statistically different from NAD alone. These data showed that cellular NAD level plays an important role as a critical factor in determining cell survival under excitotoxic injury.

### PARP-1 activation is involved in NAD depletion in NMDA-treated neurons

**Figure 2:**
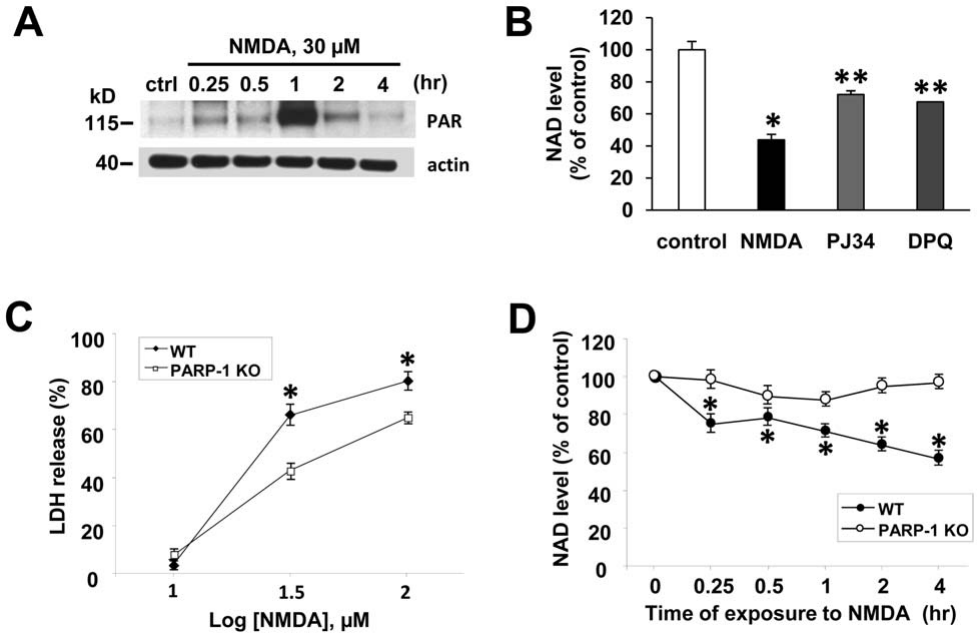
PARP-1 activation depletes cellular NAD in NMDA-treated neurons. (A) Western blot of PAR in neurons exposed to NMDA (30 µM) for indicated times. The signal of PAR peaked 1 hr after NMDA treatment. β-actin was used as a loading control. (B) NAD level was measured in neurons 4 h after exposure to NMDA alone, NMDA plus PJ34 (100 nM), or NMDA plus DPQ (25 µM) (mean ± SEM; n = 4; *P<0.01, difference from untreated control; **P<0.01, difference from NMDA alone). (C) LDH was measured in neuron cultures of WT or PARP-1 KO mice 24 h after NMDA exposure. (mean ± SEM; *n* = 8; *P<0.01, difference from the relevant controls of PARP-1 KO culture). (D) NAD level was measured in WT or PARP-1 KO cultures exposed to NMDA 30 µM for indicated times (mean ± SEM; *n* = 8; *P<0.01, difference from the relevant controls of PARP-1 KO culture).

Mounting evidence has indicated that PARP-1 is excessively activated in neurons by NMDA mediated excitotoxicity or brain ischemic injury leading to cell death by NAD depletion [13], [16], [17]. In order to verify that PARP-1 is involved in NMDA-induced NAD depletion, we examined whether PARP-1 is activated in neurons after NMDA treatment. PAR formation was significantly increased as early as 15 min after NMDA treatment, with a peak in signal 1 hr followed by a sharp decrease (Fig. 2A). Inhibition of PARP-1 with PJ 34 or DPQ significantly attenuated NAD depletion in NMDA-treated neurons (Fig. 2B). The decrease of total NAD with excitotoxic insult is consistent with our previous studies showing a decrease in total NAD with PARP-1 activation [20], and suggests that the decrease is due to depletion of cytosolic NAD and not mitochondrial NAD. In neuron cultures lacking PARP-1, neuronal death and NAD depletion with excitotoxic injury were significantly reduced (Fig. 2C and D), and confirms that activated PARP-1 consumes enormous amount of NAD inducing neuronal death. The lack of complete protection in PARP-1-deficient cells is in accord with previous reports [21], [43].

### Mitochondrial Sirt3 is increased following PARP-1-mediated NAD depletion

**Figure 3:**
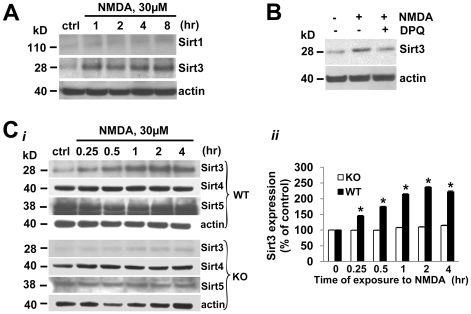
Mitochondrial Sirt3 is markedly increased in NMDA-treated neurons following PARP-1 activation. (A) Western blots of Sirt1 and Sirt3 in neurons exposed to NMDA (30 µM) for indicated times. (B) Western blot of Sirt3 in neurons 4 h after exposure to NMDA (30 µM) with or without DPQ (25 µM). (C) *i*. Western blots of mitochondrial sirtuins, Sirt3, Sirt4, and Sirt5 in WT and PARP-1 KO neurons exposed to NMDA 30 µM for indicated times. *ii*. The expression level of Sirt3 in WT and PARP-1 KO neurons was normalized with actin and quantified to the relevant control value (*t* = 0). Similar results were observed from three independent experiments (mean ± SEM; n = 3; *P<0.01, difference from control).

Next, we explored the downstream event following NAD depletion in neurons exposed to excitotoxic injury. Recently, Sirt3 as well as Sirt1 has been shown to control cellular responses to stress due to its NAD -dependent deacetylase function [44], [45], [46]. Therefore, we tested whether Sirt1 or Sirt3 serve as an important mediator for neuronal survival under excitotoxicity. We detected changes in the level of the short form (28 kD) of Sirt3 [46]. The Sirt3 antibody was verified in tissue from wt and Sirt3 knockout mice (Figure S1). The antibody recognized both the short (28 kD) and long (44 kD) forms of Sirt3 in wt tissue, which were not present in the tissue obtained from Sirt3 KO animals. Sirt3 protein level (28 kD) was markedly increased in neurons 1 hr following excitotoxic injury, whereas we found no significant change in Sirt1 protein levels with NMDA receptor activation (Fig. 3A). Since neuronal death and NAD depletion were significantly inhibited by PARP-1 inhibition, we examined the effect of inhibiting PARP-1 on the increase of Sirt3 protein levels. NMDA-induced increase of Sirt3 was attenuated by DPQ (Fig. 3B). Therefore, our data demonstrated that the increase in neuronal Sirt3 with excitotoxic injury is dependent on PARP-1 activation.

In addition to Sirt3, two other sirtuin isoforms (Sirt4 and Sirt5) were reported to localize in mitochondria. We next tested if these sirtuin isoforms were also increased with excitotoxic conditions. We examined the expressions of these mitochondrial sirtuins in neurons of WT and PARP-1 knock out (KO) mice. We found a time-dependent increase in Sirt3 levels with NMDA receptor activation in WT neurons, whereas the expression of Sirt4 or Sirt5 was not changed (Fig. 3C*i*). Furthermore, the increase in Sirt3 protein level was not found in neurons cultured from PARP-1^−/−^ mice (Fig. 3C*ii*). These data further demonstrated that the increase in Sirt3 proteins are dependent on PARP-1 activation.

**Figure 4:**
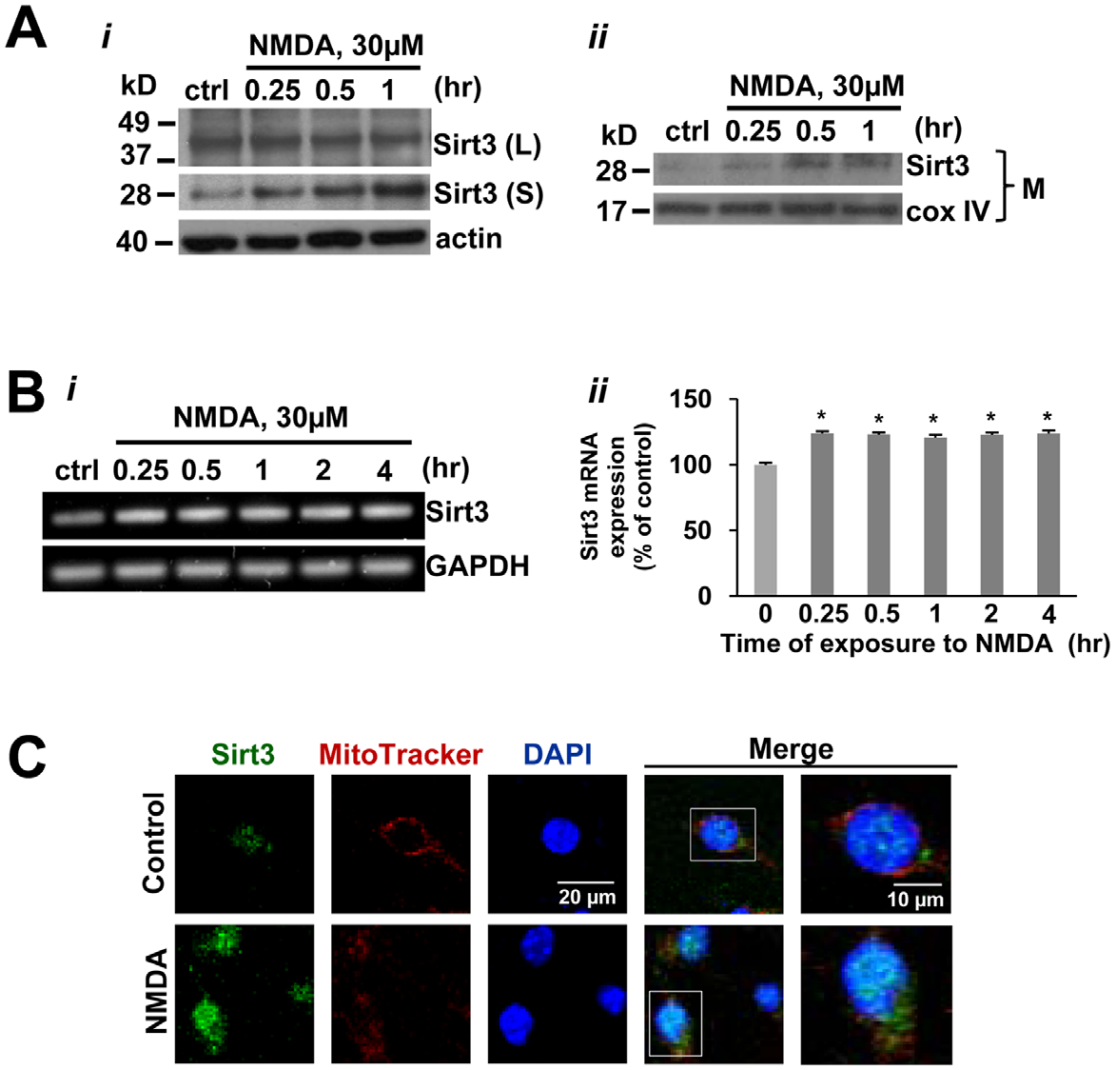
The expression and translocation of mitochondrial Sirt3 is increased in neurons after NMDA treatment. (A) *i*, Western blot of the long (L) and short (S) forms of Sirt3 in neurons treated with NMDA 30 µM at indicated times. *ii*,Western blot of Sirt3 in mitochondrial fraction (M) of neurons exposed to NMDA 30 µM for indicated times. Cox IV was used as a loading control. (B) *i*, RT PCR analysis of Sirt3 in neurons exposed to NMDA 30 µM for indicated times. *ii*, The mRNA level was normalized to the control (ctrl). Similar results were observed in three independent experiments (mean ± SEM; n = 3; *P<0.01, difference from control). (C) Confocal analysis of Sirt3 localization (green) in neurons with or without NMDA 30 µM for 4 hrs. Nucleus and mitochondria were detected with DAPI and MitoTracker red, respectively.

To determine if NMDA induces an increase in the Sirt3 long form in addition to the short form of Sirt3, we examined the protein level of both forms of Sirt3 in NMDA treated neurons. The Sirt3 long form (44 kD) was not significantly changed by NMDA while the short form of Sirt3 (28 kD) was significantly increased (Fig. 4A*i*). The increase in Sirt3 was further confirmed in mitochondrial fraction of neurons, which was detected within 15 min after exposure to NMDA (Fig. 4A*ii*). Sirt3 mRNA expression was increased in neurons by about 20% within 15 min after exposure to NMDA, which was maintained throughout the rest of the time points after NMDA exposure (Fig. 4B*i* and *ii*). Immunostaining for Sirt3 in neurons showed that Sirt3 was localized preferentially in the nucleus under basal condition, whereas it was detected in both the mitochondria as well as the nucleus after NMDA treatment (Fig. 4C). Collectively, these results demonstrated that NMDA receptor activation increased mitochondrial Sirt3 in neurons through PARP-1-mediated NAD depletion.

### Increase in neuronal Sirt3 expression and deacetylase activity is prevented by NAD treatment

**Figure 5:**
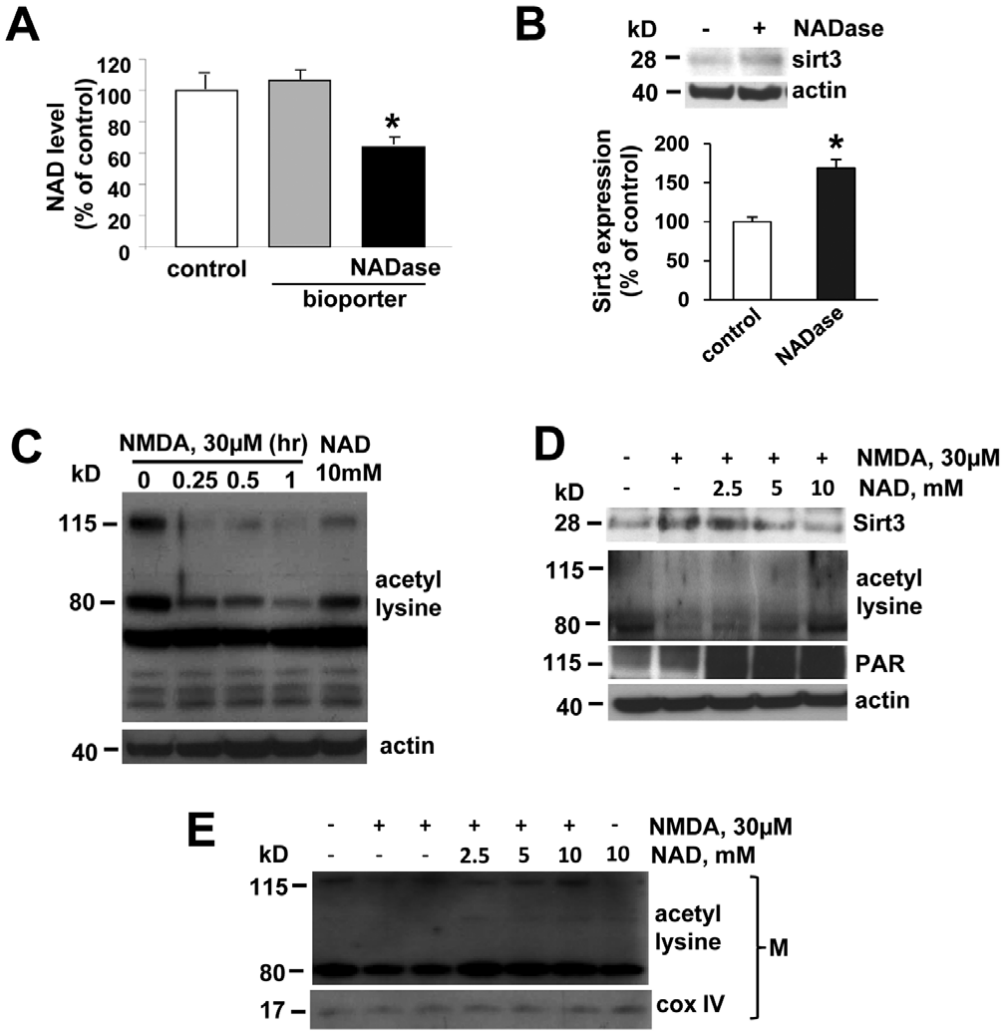
Intracellular NAD depletion causes the increased expression and activation of mitochondrial Sirt3. (A) NAD levels were measured in neurons transfected with NADase using Bioporter. NADase transfection produced a decrease in neuronal NAD content, as measured 4 hours after Bioporter tansfection (mean ± SEM; n = 4; *P<0.01, difference from control). This decrease was not observed in cultures treated with the Bioporter vehicle alone. (B) Western blot of Sirt3 in neurons with or without NADase at 4 hr after transfection.. The expression level of Sirt3 was normalized with actin and quantified to the control value (mean± SEM; n = 3; *P<0.01, difference from control). (C) Western blot of acetyl lysine in neurons exposed to NMDA 30 µM for indicated times or NAD alone. (D) The expression of Sirt3, acetyl lysine, and PAR in neurons at 4 hr after the exogenous addition of various concentrations of NAD. (E) Western blot of acetyl lysine in mitochondrial fraction (M) of neurons exposed to NMDA 30 µM, NMDA 30 µM with exogenous NAD, or NAD alone for 4 hrs.

Having demonstrated that PARP-1 activation is upstream of the increase in mitochondrial Sirt3 (Fig. 4), and having previously shown that NAD depletion was necessary and sufficient for PARP-1-mediated mitochondrial failure [21], we next tested whether the decrease in NAD was sufficient to promote Sirt3 processing. NAD glycohydrolase (NADase), an NAD catabolizing enzyme, was introduced into cultured neurons using a bioporter protein transfection method [47], a technique we have recently shown not only to decrease cytosolic NAD without PARP-1 activation (and therefore the lack of PAR formation), but also to selectively decrease cytosolic NAD and not mitochondrial NAD [21]. Introduction of NADase reduced neuronal NAD levels by about 50% relative to control and bioporter alone (vehicle control) within 4 hrs (Fig. 5A), consistent with our previous report [21]. NADase treatment of cultured neurons without bioporter transfection reagents did not decrease NAD levels (data not shown), indicating that NADase effects are not extracellular [21]. Therefore, we utilized this method to decrease cytosolic NAD independent of PARP-1 activation in order to determine if a decrease in NAD promoted an increase in Sirt3 level. NADase treatment with bioporter protein transfection increased Sirt3 protein level by 68% (Fig. 5B), indicating that NAD depletion mediated the increase in Sirt3, and suggesting that the NMDA-induced increase in Sirt3 expression is mediated by NAD depletion.

We further assayed lysine acetylation in neurons as an indirect marker for deacetylase activity. NMDA treatment induced the decrease in some deacetylase substrates with acetyl-lysine, indicating an increase in deacetylase activity in NMDA-treated neurons (Fig. 5C). The decrease in acetylation corresponded with the increase in Sirt3 protein level (Fig. 4A*i* and 5C), suggesting the involvement of Sirt3 in this increased deacetylation in NMDA-treated neurons. Because Sirt3 was increased in response to the depletion of cytosolic NAD following excitotoxic injury, we next examined whether NAD replenishment affect the expression of Sirt3 in neurons (Fig. 5D). The increase of Sirt3 along with its deacetylase activity in NMDA-treated neurons was reversed with exogenous NAD treatment in a dose-dependent manner (Fig. 5D). We demonstrated that exogenous NAD treatment protected against NMDA treatment (Fig. 1B). Here, we showed that exogenous NAD treatment increased PAR formation from NMDA treatment alone (Fig. 5D), indicating that NAD treatment promoted PARP-1 activation and suggesting that protection by NAD treatment is not due to PARP-1 inhibition. In accordance with our recent report [21], these results demonstrated that NAD depletion, and not an increase in free PAR formation, is a major cause of PARP-1-mediated neuronal death in excitotoxic injury.

A previous study demonstrated that Sirt3 is the primary mitochondrial deacetylase [48]. We confirmed the effect of NAD on Sirt3 activity by measuring acetyl lysine in mitochondrial fractions isolated from NMDA treated neurons. The level of acetyl lysine was decreased in mitochondrial fractions of neurons upon NMDA exposure, which was restored by exogenous addition of NAD (Fig. 5E). In neurons exposed to NAD alone without NMDA, the signal of acetyl lysine was not changed (Fig. 5C and 5E), suggesting no effect of NAD on Sirt3 activity under normal condition. These data indicated that NAD depletion leads to an increased expression and activity of mitochondrial Sirt3 in neurons, suggesting that Sirt3 may play a role as a critical factor in determining neuronal survival by translocating to mitochondria and affecting its function.

### Oxidative stress by NAD depletion increased mitochondrial Sirt3 expression

**Figure 6:**
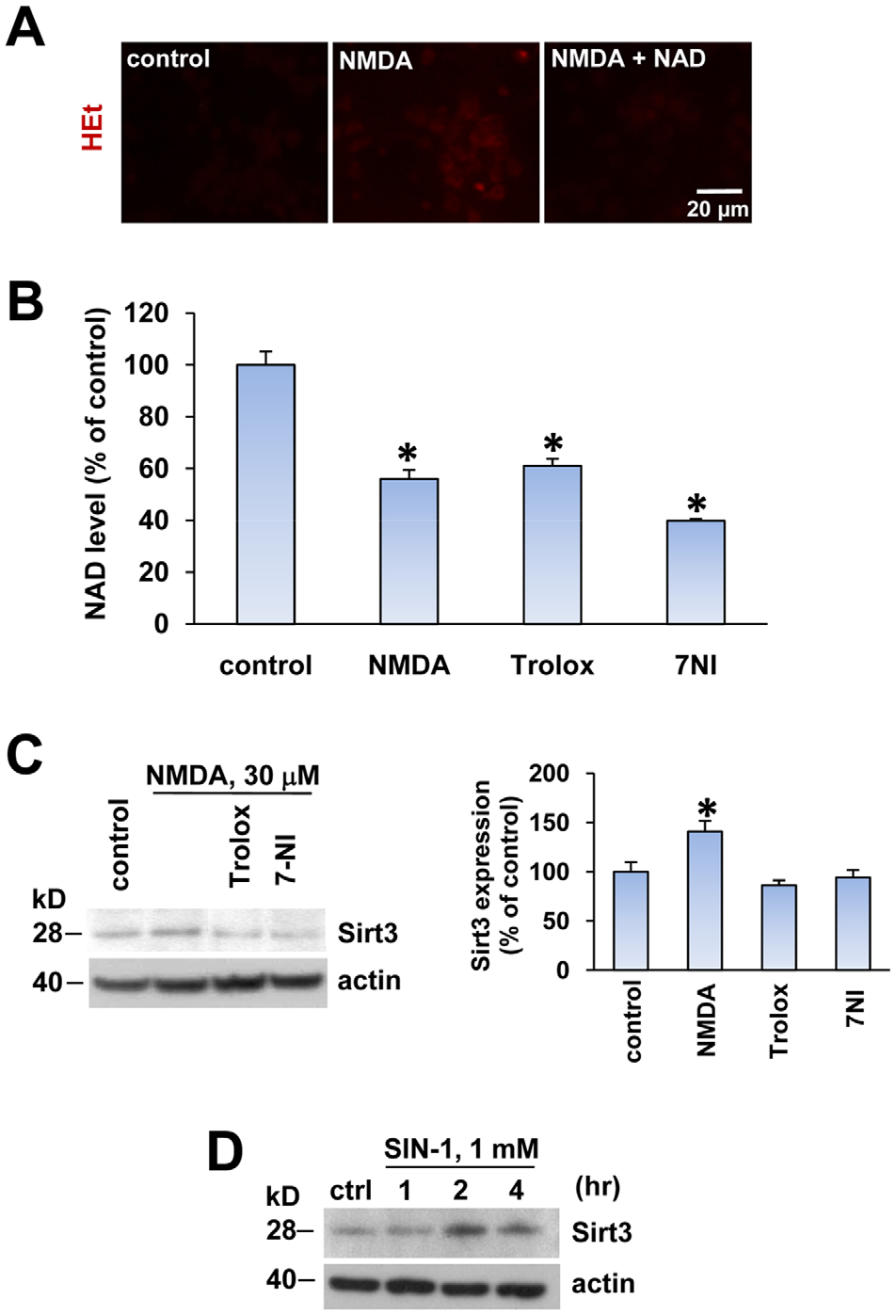
Oxidative stress by NAD depletion increased mitochondrial Sirt3 expression. (A) Fluorescence image of the oxidized dihydroethidium (HEt) in neurons 4 h after exposure to control (media exchange), NMDA (30 µM) alone, or NMDA plus NAD (5 mM). (B) Cellular NAD level was measured in neurons 4 h after exposure to control, NMDA alone, NMDA plus trolox (100 µM), or NMDA plus or 7-NI 100 nM for 4 hrs (mean ± SEM; n = 8; *P<0.01, difference from untreated control). (C) Western blot of Sirt3 in neurons treated similarly as (B). The expression of Sirt3 was normalized to actin and quantified to the control value (mean± SEM; n = 3; *P<0.01, difference from control). (D) Western blot of Sirt3 in neurons treated with SIN-1 1 mM for indicated times.

Oxidative stress has been shown to be a critical downstream event leading to neuronal death after NMDA receptor activation [49], [50], [51], [52], [53]. However, there is a lack of direct evidence in the literature demonstrating ROS-mediated PARP-1 activation with NMDA treatment. Because we demonstrated that PARP-1-mediated NAD depletion occurs in our model, we examined whether NMDA induces ROS production and therefore PARP-1 activation and NAD depletion in our excitotoxic injury model. Increased reactive oxygen species (ROS) production in neurons was evaluated by oxidation of dihydroethidium (HEt) by superoxide into the fluorescent ethidium moiety. An increase in ROS production was detected in neurons 4 h after 30 µM NMDA treatment, which was blocked by exogenously added NAD (Fig. 6A). This indicated that NAD depletion directly mediated the increase in ROS production in NMDA-treated neurons, and suggested that NAD depletion is upstream of a source of ROS production.

In order to further clarify the temporal correlation of ROS production and NAD depletion, we examined whether NAD depletion by NMDA treatement is prevented by antioxidants. We used a vitamine E analogue (Trolox) or a neuronal NOS inhibitor (7-nitroindazole, or 7-NI). Neither Trolox nor 7-NI prevented NAD depletion in NMDA-treated neurons, further suggesting that NAD depletion is an upstream event of NMDA-induced oxidative stress (Fig. 6B). Because NAD depletion induces oxidative stress, we explored the possibility that oxidative stress could be involved in the increased Sirt3. The increased expression of Sirt3 in NMDA-treated neurons was reduced by treatment with either trolox or 7-NI, which demonstrated that oxidative stress by NAD depletion directly mediates the increase of Sirt3 in neurons (Fig. 6C). In order to further test whether the increase in ROS production induces Sirt3 expression, we treated neurons with a peroxynitrite generator, 3-morpholinosydnonimine (SIN-1) (Fig. 6D). Sirt3 protein levels were increased time dependently after SIN-1 treatment, which provides a clear evidence for the involvement of oxidative stress in increased Sirt3 expression in neurons.

### Sirt3 acts as a prosurvival factor in neurons under excitotoxic injury

**Figure 7:**
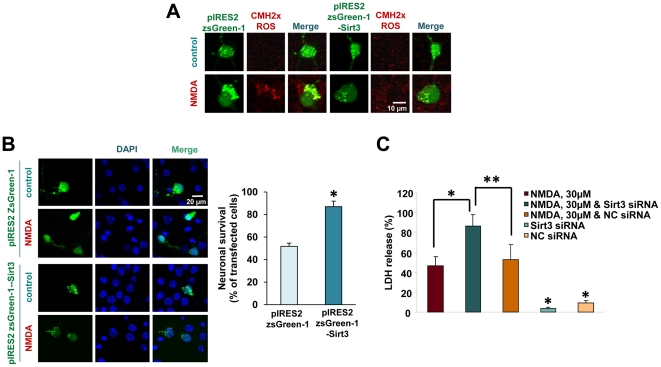
Sirt3 acts as a prosurvival factor against excitotoxic injury. (A) Fluorescence image of mitochondrial ROS detected with CMH2xROS in neuron cultures transfected with pIRES2-zsGreen1 (control vector) or pIRES2-zsGreen1-Sirt3 at 24 hrs after exposure to NMDA 30 µM. (B) The expression of control vector alone and pIRES2 ZsGreen-1-Sirt3 in transfected neurons 24 hr after exposure to NMDA 30 µM. Cell death was analyzed by counting the pyknotic bodies (shown as DAPI) in transfected neurons (mean ± SEM; n = 8; *P<0.01, difference from the culture transfected with the control vector). (C) Cell death was analyzed by LDH release in neurons transfected with Sirt3 siRNA or negative control siRNA at 24 hrs after exposure to NMDA 30 µM (mean ± SEM; n = 8; *P<0.01, difference from NMDA alone; **P<0.01, difference from NMDA plus Sirt3 siRNA).

Recently, the protective effect of Sirt3 under oxidative stress was shown in cardiomyocytes [40], where it was shown that Sirt3 reduced ROS generation in cardiomyocyte under cardiac hypertrophy by enhancing antioxidant enzymes such as manganese superoxide dismutase (MnSOD) and catalase. Indeed, our evidence is in support of these findings (Fig. 6). To examine whether Sirt3 plays a protective role in neurons, we observed mitochondrial ROS generation in neurons transfected with pIRES2 ZsGreen-1 with or without Sirt3 at 24 hrs after NMDA treatment. In neurons overexpressing Sirt3, mitochondrial ROS generation was significantly inhibited compared with in neurons expressing control vector only, implicating the protective role of Sirt3 in neurons (Fig. 7A). To further explore the protective role of Sirt3 in neurons under excitotoxic injury, we examined neuronal viability by staining neurons with Hoechst and counting the number of pyknotic neuron bodies in cultures overexpressing Sirt3 or control vector 24 hrs after NMDA treatment. Neuronal death was significantly reduced in cultures transfected with Sirt3, suggesting a critical role of Sirt3 in neuronal survival under stress condition (Fig. 7B). To confirm the prosurvival effect of Sirt3, we knocked down Sirt3 in neurons using Sirt3 siRNA. Knockdown of Sirt3 exacerbated excitotoxic neuronal death, whereas neurons transfected with negative control siRNA showed no difference in NMDA-induced neuronal death from the cultures without transfection (Fig. 7C). These results showed the robust evidence of the pro-survival role of Sirt3 in neurons under excitotoxic stress.

## Discussion

Mitochondrial localization of Sirt3 plays a role in various mitochondrial functions, such as maintaining basal ATP level and regulating apoptosis. Sirt3 has been shown to regulate energy homeostasis [38]. Recent studies have suggested the protective role of Sirt3 in cells under stress condition. In this study, we i) identified the presence of Sirt3 in neurons, and ii) demonstrated that Sirt3 plays a role as a prosurvival factor in neurons against excitotoxic injury. We found that under excitotoxic injury, intracellular depletion of NAD with induced increased expression of Sirt3 in mitochondria. NAD depletion was induced either by PARP-1 activation, or by protein transfection of NADase, both of which have been shown by our group to selectively decrease cytosolic NAD [19], [20], [21]. ROS produced following PARP-1 mediated NAD depletion was involved in the increased expression of Sirt3. Overexpression of Sirt3 reduced NMDA-induced ROS generation, thereby preventing neuronal death, whereas knockdown of Sirt3 enhanced neuronal death. This study is the first evidence demonstrating that the increase in mitochondrial Sirt3 contributes to neuroprotection from excitotoxicity.

Excessive activation of PARP-1 induces neuronal death under stress conditions such as DNA damage, excitotoxicity, or oxidative stress [17], [54]. PAR has been shown to induce mitochondrial release of AIF and cell death [15], [55]. However, our previous studies have shown that NAD depletion is the cause of PARP-1-mediated cell death and exogenous addition of NAD effectively inhibited cell death [19], [21], [56]. Moreover, we have shown that activated PARP-1 depleted most of the cytosolic NAD and then consumed the mitochondrial NAD only after MPT permits mitochondrial NAD to exit into the cytosol [19], [20]. In accordance with our previous findings, this study demonstrated that PARP-1- mediated NAD depletion is responsible for neuronal death with chronic exposure to low-dose excitotoxic agents [21], [57]. Addition of exogenous NAD is a very novel concept, yet proving to be very effective [11], [19], [21]. We demonstrated that NAD uptake into cells is mediated by P2X7 receptor-gated channels [21]. Activation of P2X7 receptors occurs under basal conditions, and P2X7 receptors are further activated in injury conditions and allows for an increase in permeability. Therefore, it is not surprising that NAD levels are further increased under excitotoxic conditions. In addition, we have unpublished data suggesting that NAD uptake into cells is increased when cells are exposed to oxidative injury. In this study, intracellular NAD level was higher in neurons treated with NAD in the presence of NMDA than those treated with NAD alone (Fig. 1C).

**Figure 8:**
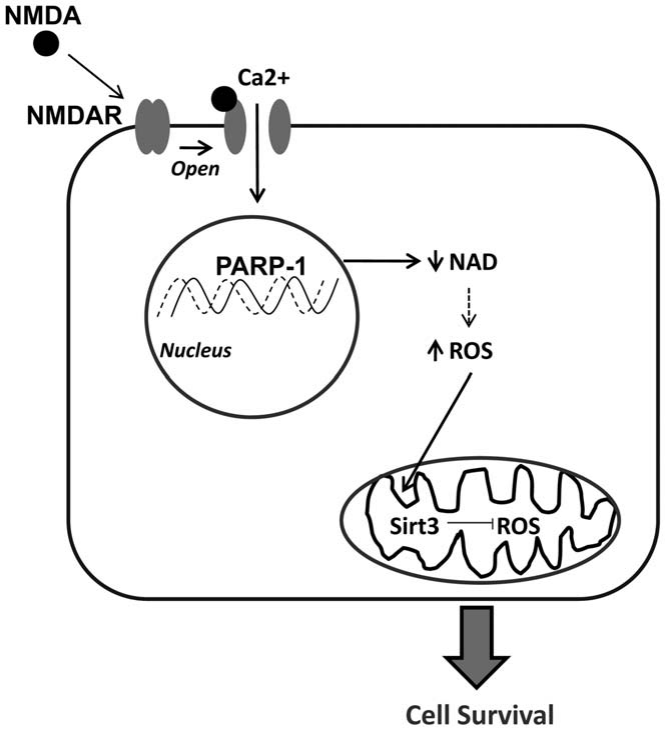
Schematic Diagram of NMDA-induced increase in mitochondrial Sirt3. The proposed mechanism showing the role of Sirt3 in protecting against NMDA-induced excitotoxic injury. NMDA binds to the NMDA receptor which opens the ion channel and allows for calcium (Ca^2+^) influx into the cell and promotes oxidative stress. Oxidative DNA damage activates PARP-1 and decreases NAD, leading to an increase in ROS production due to mitochondrial failure. The increase in ROS promotes an increase in mitochondrial Sirt3. Mitochondrial Sirt3 levels were increased in NMDA-treated neurons following PARP-1 mediated NAD depletion. Further evidence is provided to support this hypothesis through genetic modification: Overexpression of Sirt3 prevented the increase in ROS production and neuronal death, whereas knock down of Sirt3 exacerbated neuronal excitotoxic injury.

In the present study, we demonstrated that cytosolic NAD decreased rapidly in neurons within 4 hr after NMDA treatment, yet mitochondrial NAD levels remained unchanged. This is consistent with previous findings that PARP-1 activation selectively depletes cytosolic NAD prior to mitochondrial NAD release and utilization for PAR formation [20], [21]. In support of this, it was found that Sirt3 functioned as a mitochondrial NAD -dependent deacetylase [58], and the results from our findings imply that the NAD -dependent deacetylase activity of Sirt3 in mitochondria is maintained due to the preservation of mitochondrial NAD, whereas Sirt1 deacetylase activity is impaired due to depletion of cytosolic NAD. Consistent with our study, it was shown that maintenance of mitochondrial NAD under stress condition can promote cell viability even though cytosolic and nuclear NAD is depleted [59]. Our studies support this finding, since we also demonstrate that conditions that promote intact mitochondria (and therefore intact mitochondrial NAD levels) prevents neuron death, which we similarly reported in MNNG-treated neurons [20] as well as in ischemia/reperfusion injury in cardiac myocytes [22]. However, one major difference may lie in the hypothesis. Yang et al demonstrated that mitochondrial NAD levels regulate cell survival (independent of changes in cytosolic NAD), whereas we demonstrated that cytosolic NAD depletion preceded mitochondrial NAD depletion, and that mitochondrial NAD depletion was mediated by opening of the mitochondrial permeability transition pore and release of the mitochondrial NAD pool [20], [21]. Interestingly, our data shows that mitochondrial NAD levels increase after NMDA treatment. The reason for this is still unclear, and remains to be further studied. One possible explanation is that the increase in mitochondrial NAD level plays a significant role in promoting cell viability, and this hypothesis supports the findings by Yang *et al* [59]. Cellular NAD levels were restored by addition of exogenous NAD, which prevented excitotoxic neuronal death in spite of an enhanced generation of PAR polymers. Our results show clear evidence that cytosolic NAD depletion directly contribute to excitotoxic neuronal death, and we outline our working hypothesis as a schematic diagram in Figure 8.

Since the identification and characterization of sirtuins in several age-related diseases including neurodegenerative diseases, modulation of sirtuin activity as a therapeutic target for prevention and cure of such diseases has received lots of interest. Sirt1 has been extensively studied relative to the other Sirt isoforms. Resveratrol is known to be an activator of Sirt1 *in vitro*, and the mechanism of activation has been demonstrated [60], [61]. Although the role of sirtuins in neuronal survival has been recently documented, the exact mechanism of neuroprotection by sirtuins is still unclear and somewhat controversial. A recent report has shown that Sirt1 is upregulated in mouse models for Alzheimer's disease and amyotrophic lateral sclerosis protecting against neurodegeneration [35], whereas another report showed that Sirt1 increasingly expressed in neurons by excitotoxic injury induces NAD depletion enhancing neuronal death [9]. Further, it was shown that Sirt1 protected neurons from low potassium-induced apoptosis [62], while Sirt2, Sirt3 and Sirt6 induced apoptosis and Sirt5 had protective effect depending on the subcellular localization. In the present study, we reported that only Sirt3 was markedly increased in neurons upon NMDA induced excitotoxicity, whereas no changes in Sirt1 or other mitochondrial Sirts (Sirt4 or 5) were detected with excitotoxic injury.

The exact sequence of events involved in Sirt3 processing and translocation to mitochondria is still unclear because of conflicting data in the literature. For example, it was reported that Sirt3 is processed in the nucleus prior to translocation to mitochondria [37], [63], whereas another group reported that the long form is processed in the mitochondria to the short form [58]. In this study, immunofluorescence of Sirt3 detected by an antibody specific to short Sirt3 was increased in both the nucleus and mitochondria in NMDA-treated neurons, suggesting that the increase in Sirt3 by excitotoxic conditions may occur both within and outside of mitochondria. Also, what is unclear is our reported Sirt3 long form size of 44 kD, which is different from the long form size (35 kD) reported by others [64], [65]. Wherever Sirt3 may be processed, our finding presented here demonstrated that excitotoxicity induces the increased expression and translocation of Sirt3 into mitochondria in neurons.

Recent evidence demonstrated the crucial role of NAD as a determining factor in cell survival under energetic stress, and the role of Sirt3 as a metabolic sensor is gaining further interest. Sirt3 was reported either in mitochondria [66], [67] or was shown to translocate from the nucleus to mitochondria [63], and Sirt3 expression and/or processing has been shown to increase in various tissues during stress conditions [37]. Prior studies showed the role of Sirt3 in maintaining energy metabolism. The role of Sirt3 in the maintenance of ATP level and in regulating mitochondrial electron transport was shown using Sirt3 knockout mice [38]. It was reported that constitutive expression of Sirt3 promoted the expression of mitochondrial genes, leading to enhanced mitochondrial electron transport activity [37]. Interestingly, we found that NAD depletion by NADase protein delivery increased expression of mitochondrial Sirt3, whereas exogenous treatment with NAD prevented the increase in Sirt3 and rescued neurons. Our finding seems contradictory to a recent report that showed the activation of Sirt3 by exogenously added NAD [11]. However, both studies demonstrated that exogenously added NAD rescued cells. It is likely that cytosolic NAD depletion induced mitochondrial Sirt3 to protect neurons from further energetic stress. Similar to our findings, Hirschey *et. al.* showed that Sirt3 expression was upregulated during fasting in liver and brown adipose tissue and modulated mitochondrial fatty acid use [68]. Our study suggested that Sirt3 could play a pivotal role in protecting neurons from excitotoxicity-mediated energetic stress by showing that Sirt3 was increased and activated in mitochondria, another pool of NAD, upon cytosolic NAD level depletion.

Excitotoxicity induced by excessive calcium influx into neurons initiates ionic imbalance, oxidative stress, and energy failure. Exogenously added NAD inhibited ROS generation in neurons treated with NMDA, whereas antioxidant agents such as trolox or 7-NI could not reduce NAD depletion. Once NAD turnover is enhanced by NMDA receptor activation, it seems that the treatment with antioxidants is not sufficient to prevent NAD depletion. However, BAPTA-AM, a cell-permeable Ca^2+^ chelator, significantly prevented NAD depletion (data not shown), indicating that Ca^2+^-dependent intracellular signal transduction pathways may play an important role in this proposed mechanism. This data further suggests that other events following excessive deregulated [Ca^2+^]_c_ increase may also contribute to NAD depletion. For example, we previously reported that NMDA receptor activation promoted MPT induction presumably through excessive mitochondrial Ca^2+^ uptake, and MPT induction promoted release of mitochondrial Ca^2+^ and contributed to deregulated [Ca^2+^]_c_ increase [69]. In addition to reducing ROS generation, exogenous addition of NAD almost completely prevented neuronal death, suggesting the definitive role of NAD in inhibiting the above events by excitotoxicity. Interestingly, we found that Trolox or 7-NI reduced Sirt3 expression. Consistent with this result, we observed that Sirt3 was increased in neurons treated with the peroxynitrite generator. These observations suggest that in our excitotoxicity model, NAD depletion is an upstream event of oxidative stress, and seems to be primarily responsible for the increased expression of Sirt3 in mitochondria.

What we posit is that the normal cell response to pathological insults is to reverse this stress. In this model, excitotoxic injury through opening of NMDA receptor-gated calcium channels increases ROS formation through PARP-1 activation and NAD depletion, which promotes an increase in mitochondrial Sirt3 to reduce further ROS formation (such as ROS-induced ROS formation). An increase in mitochondrial Sirt3 sufficient to counter further ROS formation will prevent injury, and inadequate mSirt3 will promote pathological ROS formation and subsequent cellular demise. Thus, a delicate balance exists to promote either cell survival (increase in mSirt3) or cell death (increase in ROS), depending on the degree of response. This is described in our schematic diagram (Fig. 8).

The role of Sirt3 in regulating ROS production has been demonstrated in recent studies. Constitutively expressed Sirt3 was shown to reduce ROS in adipocytes [37], and the increased expression of Sirt3 protected myocytes from genotoxic and oxidative stress and blocked cardiac hypertrophy by activating antioxidant enzymes such as MnSOD and catalase [40], [46]. Recently, Sirt3 knock out mouse embryonic fibroblast was shown to exhibit increased superoxide level [70]. In support of these findings, we found that increased expression of mitochondrial Sirt3 significantly reduced ROS production following excitotoxic injury, whereas Sirt3 silencing increased ROS production. Neurons transfected with Sirt3-overexpressing plasmid showed less mitochondrial ROS production following excitotoxic injury. Our finding implies that the expression of Sirt3 in mitochondria was increased in response to oxidative stress following excitotoxic injury, which is required for neuroprotection against oxidative stress-mediated death. Moreover, the overexpression of Sirt3 inhibited excitotoxicity-mediated neuronal death, whereas Sirt3 siRNA transfection exacerbated neuronal death. The exact mechanism of how Sirt3 decreases mitochondrial ROS production in neurons is unclear and remains to be further investigated. Since the role of Sirt3 in neuronal survival has not been established, our study provides the first evidence that Sirt3 plays a pivotal role in neuronal survival under stress condition such as excitotoxic injury.

In conclusion, we have shown that increased Sirt3 in mitochondria plays a pivotal role in neuroprotection against NMDA induced excitotoxicity, and sheds light into the effects of Sirt3 on the regulation of neuronal survival. We demonstrated that NAD depletion and subsequent oxidative stress induced the increase of Sirt3 in neurons under NMDA-mediated oxidative stress. Since oxidative stress and energetic failure contribute to neurodegeneration, the roles of Sirt3 in controlling ROS generation and maintaining energy homeostasis have profound implications for intervention of neuronal death by neurodegenerative diseases.

## Materials and Methods

### Materials

3,4-dihydro-5-[4-(1-piperidinyl)butoxy]-1(2H)-isoquinolinone (DPQ) and PJ34 were obtained from Calbiochem (Gibbstown, NJ). MitoTracker, DAPI, dihydroethidium (HEt), CMH2xROS, and 3-morpholinosydnonimine (SIN1) were obtained through Invitrogen (Carlsbad, CA). All other reagents were purchased from Sigma Chemical (St. Louis, MO) except where otherwise noted. The studies were approved by the San Francisco Veterans Affairs Medical Center (SFVAMC) animal studies committee.

### Mice

PARP-1-deficient mice [13] were generated from targeted 129S2/SvPas-derived D3 ES cells in isogenic C57BL/6 mice (genetic designation 129S-Parp1^tm1Zqw^/J, JAX stock number 002779) using a targeting vector containing a neomycin resistance gene to disrupt exon 2 of the parp gene. The chimeric animals obtained were backcrossed for 8–12 generations with wild-type (wt) littermate C57BL/6 mice to generate PARP-1 knockout C57BL/6 mice. PARP-1 knockout mice were backcrossed for 8–12 generations with CD-1 mice to obtain F3 CD-1/C57BL/6 mice in order to generate PARP-1 knockout PARP-1^−/−^ CD-1 mice. Heterozygous (PARP1^+/−^) mice were then bred to obtain litters consisting of homozygous (PARP-1^−/−^), heterozygous (PARP-1^+/−^), and wt siblings. Homozygous PARP-1^−/−^ and wt mice were identified by PCR genotyping. All animals were maintained in specific pathogen-free conditions according to the regulation of the SFVAMC animal studies committee. Tissues from Sirt3 deficient mice were contributed by Dr. Gary Cecchini, and the mice originated from Dr. Frederick Alt's group.

### Primary neuron culture

An animal protocol for ethical use of animals for research was approved by the San Francisco Veterans Affairs Medical Center animal studies committee (ACORP Nos. 07-040-03 and 07-010-03). The investigation conforms to the Guide for the Care and Use of Laboratory Animals published by the US National Institutes of Health (NIH Publication No. 85-23, revised 1996). Cerebral cortical cultures were prepared as previously described [20], [21], [57] from 15-day mouse embryos in MEM medium (Invitrogen) containing 2 mM glutamine, 10% FBS, and 1% penicillin and streptomycin. A colony of PARP-1^−/−^ mice [13] and the wt littermates were maintained on-site. On DIV (days in vitro) 2, 10 µM cytosine arabinoside was added for 24 h to prevent glial proliferation. The neurons were subsequently maintained in serum-free neurobasal medium (Invitrogen) containing 2% B27 supplement and 2 mM glutamine at 37°C in a humidified 5% CO_2_ incubator, and were given fresh media every 3–4 days. Experiments were conducted on DIV 9–10 by exchanging the culture medium with Hank's balanced salt solution (HBSS) containing 1.2 mM CaCl_2_, 0.8 mM MgSO_4_ (Invitrogen).

### NAD assay

NAD assay was performed as previously described [20], [21], [57]. Neurons were extracted in 0.5 N HClO_4_, neutralized with 3 M KOH/125 mM Gly-Gly buffer (pH 7.4), and centrifuged at 10,000× *g* for 5 min. Supernatants were mixed with a reaction medium containing 0.1 mM 3-[4,5-dimethylthiazol-2-yl]-2,5-diphenyl-tetrazolium bromide (MTT), 0.9 mM phenazine methosulfate, 13 units/ml alcohol dehydrogenase, 100 mM nicotinamide, and 5.7% ethanol in 61 mM Gly-Gly buffer (pH 7.4). The A_560 nm_ was determined immediately and after 10 min, and results were calibrated with NAD standards. Results were normalized to protein content as determined by the bicinchonic acid (BCA) method. The NMDA-induced NAD decrease was compared among the different experiments and not found to be statistically significant.

### Neuronal death

Between days 9 and 10 in vitro (DIV 9–10), cultures were rinsed with HBSS and treated for 24 h with 30 µM NMDA, with or without other drugs. Cell death was quantified as previously described [21] by measuring lactate dehydrogenase (LDH) release into the bathing medium over 24 h and was expressed as a percentage of cell death induced by a maximally cytotoxic concentration (500 µM) of NMDA: (LDH−LDH_control_)/(LDH_NMDA_−LDH_control_)×100%. Difference in NMDA-induced neuronal death among the different experimental groups were compared and not found to be statistically significant.

### Western blot analysis

Western blots were prepared as described [21]. Neuron cultures were lysed and collected in RIPA buffer (Cell signaling) with 1 mM PMSF on ice for 30 minutes. Cell lysates were centrifuged at 14,000 g for 10 minutes, and cell extracts were mixed with a 1∶4 volume of SDS–PAGE loading buffer (10% β-mercaptoethanol, 10% glycerol, 4% SDS, 0.01% bromophenol blue, and 62.5 mM Tris–HCl, pH 6.8) and heated to 65°C for 15 min. Samples were loaded on a 10% resolving SDS–polyacrylamide gel, and transferred to polyvinyldifluoridine membranes. Membranes were incubated overnight at 4°C with rabbit polyclonal anti-Sirt1, anti-Sirt3, anti-Sirt5, goat polyclonal anti-Sirt5 (1∶500; Abcam), rabbit polyclonal anti-Acetyl-lysine (1∶1,000; Biomol), rabbit polyclonal anti-PAR (1∶1,000; Trevigen) or rabbit polyclonal anti-β-actin (1∶5,000; Abcam) antibodies, and then reacted with anti-rabbit or anti-goat secondary antibodies (1∶10,000; Vector Laboratories). Immunoreactivity was detected with luminol reagent (GE).

### Mitochondrial Isolation

Mitochondria were isolated as described previously [21], using mitochondrial isolation kit (Thermo Fisher Scientific) according to the manufacturer's protocol. Cells were grown on 6-well plates as described, and were harvested with a scraper in 0.5 mL of isolation medium (320 mM sucrose, 1 mM potassium EDTA, and 10 mM Tris–HCl pH 7.4). Cells were homogenized in a glass Teflon homogenizer, and were centrifuged at 500×g for 5 min at 4°C. The supernatant fractions (S1) were saved (at 4°C) for the subsequent centrifugation step. The pellet was re-homogenized and centrifuged at 500×g for 10 min at 4°C. The supernatant (S2) was combined with S1 and centrifuged at 500×g for 10 min at 4°C. The supernatant was collected, and centrifuged at 16,000×g for 30 min. The final pellet (mitochondrial fraction) was resuspended in 200 µl of isolation medium and kept on ice for biochemical studies. Mitochondrial enrichment was quantified by western blots using an antibody targeted to the mitochondrial protein cytochrome C oxidase IV (cox IV).

### RT PCR analysis

Total RNA was isolated using Trizol reagent (Invitrogen). First strand cDNA synthesis was performed using Superscript II reverse transcriptase primed with oligo dT primers (Invitrogen). PCR was performed in 50 µl volumes under the following condition; 94°C for 2 min, followed by 30 cycles of 94°C for 30 sec, 55°C for 30 sec, and 72°C for 45 sec, and 72°C for 10 min. Sense and antisense primers were: GCCTGCAAGGTTCCTACTCC and TCGAGGACTCAGAACGAACG for Sirt3; TCCATGACAACTTTGGCATCGTGG and GTTGCTGTTGAAGTCACAGGAGAC for GAPDH. PCR product was analyzed using LAS-4000 luminescent image analyzer (Fuji).

### Immunocytochemistry

Cortical neurons were fixed in 4% paraformaldehyde for 10 min and immunostained using anti-goat Sirt3 antibody (1∶200 dilution; Santa Cruz) at 4°C for overnight. Immunostaining was visualized with Alexa Fluor 488–conjugated antibody (1∶400 dilution; Invitrogen). Cell fluorescence was captured using a Zeiss LSM 510 META confocal laser scanning module mounted onto an inverted Axio Observer (Zeiss), using a 40× EC Plan-NEOFLUAR (NA 1.3) oil objective. Data was acquired with the Zeiss LSM 510 AIM software. All imaging was performed at RT.

### NAD glycohydrolase transfection

NAD glycohydrolase (NADase; Sigma, St. Louis, MO) protein transfection was performed as previously described [21]. Briefly, NADase was diluted to 1–5 mg/ml in PBS, and NADase was diluted in HBSS to the proper working concentrations (1–500 µg/mL) as determined after calculating the activity units, and mixed with BioPorter reagent (Gene Therapy Systems, San Diego, CA) and the BioPORTER-protein complex was subsequently diluted in HBSS. The cell cultures were incubated with the BioPORTER-protein complex for 3–4 hr at 37°C and immediately used for experiments. Positive controls (fluorescein-labeled antibody and β-galactosidase) were used to verify protein transfection (data not shown).

### ROS production

ROS generation was assayed with minor modifications using chloromethyl derivative of dihydro X-rosamine (or CM-H_2_XROS; Invitrogen), a modified version of MitoTracker Red, and dihydroethidium (HEt; Invitrogen, [71]. Cultures were loaded with 5 µM HEt or 300 nM CM-H_2_XROS in HBSS for 20 min at 37°C and washed 3 times with HBSS. The image of ROS was captured at 37°C using a Zeiss LSM 510 META confocal laser scanning module mounted onto an inverted Axio Observer (Zeiss) (for HEt excitation = 546 nm and emission = 590 nm; for oxidized CM-H_2_XROS, excitation = 580 nm and emission = 600 nm).

### Constructs of Sirt3- expressing vector

Total RNA was isolated from cultured mouse primary cortical neurons and used for RT-PCR to produce the coding region of mouse Sirt3. First strand cDNA synthesis was performed with1 µl of total RNA as template using Superscript II reverse transcriptase (Invitrogen). The DNA sequences of Sirt3 primers with restriction enzyme sites for cloning were as follows - forward primer with EcoRI site; 5′-CCGGAATTCCGGATGGTGGGGGCCGGCATC-3′, reverse primer with XmaI site; 5′-CCCCCCGGGGGGTTATCTGTCCTGTCCATCCAGCTT-3′ (Integrated DNA technologies). The PCR was performed under the following condition: 94°C for 2 min, followed by 40 cycles of 94°C for 30 sec, 58°C for 30 sec, 68°C for 1 min 30 sec. The Sirt3 coding sequence produced by PCR was cut by EcoRI and XmaI, and inserted into pIRES2-zsGreen1vector (Clontech) for construction of pIRES2-zsGreen-Sirt3. pIRES2-zsGreen1 with no insert was used as control vector. The pIRES2-zsGreen vector was only used to label cells that transfected successfully, and not to label Sirt3 localization.

### Transfection of primary cortical neurons

For transfection of primary neurons Neon transfection kit (Invitrogen) was used according to the manufacturer's instructions. Primary cortical neurons collected from 15 day CD1 mouse embryos were prepared at final concentration of 5×10^7^/ml in resuspension buffer (Invitrogen), 10 µl of which per well in 24 well plate was transfected with 0.5 µg of vector or 100 nM of Sirt3 siRNA (Ambion) at 1500 V with two pulses of 10 ms. Transfected neuron cultures were used on DIV 8–10.

### Data Analysis

Data are presented as means ± SEM. Statistical significance was assessed using ANOVA and the Student-Newman-Keuls *post hoc* test to compare the indicated experimental groups. The “n” denotes the number of independent experiments, each performed with independently prepared cultures. Values from 2–4 culture wells were averaged to generate each data point within each experiment. *P*<0.01 were considered significant.

## Supporting Information

Figure S1 Detection of Sirt3 by immunoblotting in brain tissue from Sirt3 WT and KO mice. Sirt3 proteins at 44 kDa and 28 kDa were detected in WT but not in Sirt3 KO mice brain tissues.
(0.81 MB TIF)
