# Supplementary figures and images for: Neuronal Sirt3 Protects against Excitotoxic Injury in Mouse Cortical Neuron Culture
Source: PLoS One. 2011 Mar 1;6(3):e14731. doi: 10.1371/journal.pone.0014731 (PMC3046953; doi:10.1371/journal.pone.0014731)

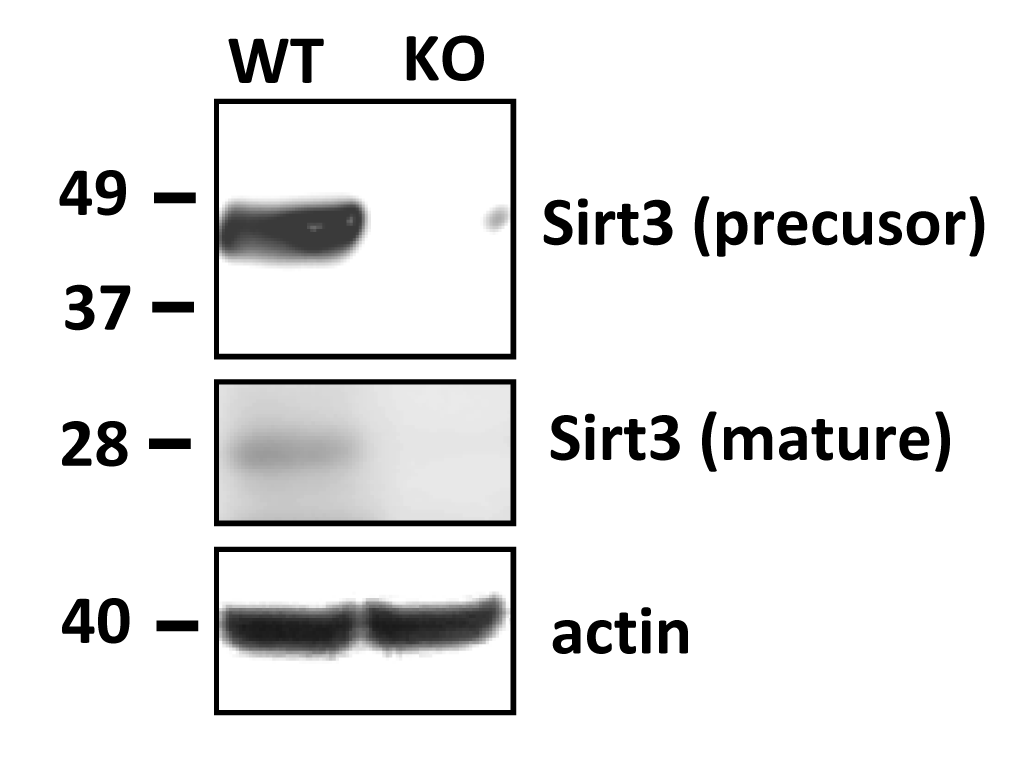

Supplement: Figure S1 — Detection of Sirt3 by immunoblotting in brain tissue from Sirt3 WT and KO mice. Sirt3 proteins at 44 kDa and 28 kDa were detected in WT but not in Sirt3 KO mice brain tissues. (0.81 MB TIF) [file pone.0014731.s001.tif]
